# A Dissertation on the Practice of Medicine; Containing an Account of the Causes, Symptoms, and Treatment of Diseases, and Adapted to the Use of Physicians and Families

**Published:** 1849-09

**Authors:** 


### BIBLIOGRAPHICAL NOTICES.

*A Dissertation on the Practice of Medicine; containing an account of the Causes, Symptoms, and Treatment of Diseases, and adapted to the use of Physicians and Families.* By Tomlinson Fort, M. D., Milledgeville, Ga., 1849. 8vo. pp. 740.

In acknowledging the receipt of this volume, we are somewhat at a loss, notwithstanding the title, to determine in what catalogue to place it. According to the dedication, it is an " attempt to give the Science of Medicine a wider range in the mental operations of the age," in the shape of a " Dissertation on the Practice of Medicine." In his advertisement, the author confesses that a great object with him was to present himself " to the best advantage before the Medical Profession."

His manner of exercising this laudable ambition is, to say the least of it, a singular one, and altogether novel in this particular relation. The means he has adopted, however, with sorrow be it said, are now-a-days only too familiar; not in the hands of truly scientific men, but in those of the very charlatans whom his book was put forth to overwhelm, and whose boast it is to stand ever worst instead of best in the esteem of legitimate practitioners and teachers. But more of this directly.

The author informs us further, in the same advertisement, that his "work is in its nature ephemeral," and that he "does not claim for it a place among the standard works of the day." And whilst he tells us, what is sufficiently apparent, that he has made very few acknowledgments to authors, he very generously hints, that should the reception of his bantling "warrant it, he hopes to live to remedy some of its defects, and especially to do justice to the labors of others."

We are disposed to be duly thankful for these good intentions, and freely join with him in the hope of his surviving for the task; but we quite as much regret that he has not taken the time and trouble to apply the remedy, and do the justice promised while time was yet his own, were it only for the benefit of those into whose hands the first edition is to fall. We say this in all sincerity and seriousness; for apart from the absolute inadmissibility of the stereotyped excuse (not offered in the present instance) of the pressing occupations of an extensive practice, &c., &c., in an avowedly systematic work, whether ephemeral or not, we think that for the sake of the unprofessional readers to whom the "dissertation" is in part addressed, if not for that of the highly respectable body of gentlemen to whom it was dedicated, it should not have been allowed to appear without a far more complete revision than it seems to have received.

Although written in a generally clear, correct and sometimes forcible style, it is sadly disfigured with errors of medical orthography, if not of erudition; and we honestly believe that if the importance of emendation and correction is to be the motive for a new edition, the sooner the first is exhausted, the better it will be for all concerned.

If there were reason to doubt the character of the work before us, the advertisement above alluded to would dissipate it at once. The grand design is to illuminate the country, and thus to make an impression on "the age," which shall redeem and disenthrall the human family from the shackles of quacks and quackery, and drive the whole herd out of every field, by rendering, henceforth and forever, "every man *their* own doctor."

This may be a novel and impressive enterprise in the author's own locality, although we are loth to believe that any State of the Union is yet so far behind the times. At any rate, we understand that the same thing has been tried here and elsewhere on this side of the Atlantic, and may be tried again. We are also under the impression that it has occasionally been thought of among the more sophisticated people of the old world; but we have yet to learn that the mixture, pill and powder business has suffered much decline therefrom; or that Catholicons and Panaceas, Poor Man's Plasters, or any of the whole range of the *Materia Medica Arcana* have lessened in demand or ceased in giant growth.

To use the Doctor's own words, after stating his desire to present himself well before his professional brethren, he says:

"It was my purpose to shew them, that the time had arrived, when a diffusion of medical knowledge among men, was necessary to the continuance of their confidence in the science, or its professors; that the decline of that confidence which is manifest in this day of increasing civilization, has arisen from the vain attempt to make medicine an exclusive property in the hands of those who pursue it as a profession; that the belief of every preposterous theory so readily entertained, and the sport with human life now carried on so widely under courses of treatment as opposite as they are destructive, are cherished and kept up by the same cause; that instead of putting down the quack, this exclusive system is the rampart behind which he stands in safety, and makes more by the random fling of ignorance and rapacity, than the most gifted of his adversaries by years of study and labor; and, finally, that it would be greatly to the credit of medical men, and profitable to the whole profession, to throw open the doors of their science, and by all practicable means induce mankind to consider it their duty to obtain some knowledge of it for themselves."

This is the sum and substance of an introduction for which the advertisement was substituted, and which was nipped in the bud by an attack of illness of the author.

This has proved an unfortunate extinguisher, inasmuch as a fuller explanation of his views would have given us, perhaps, a more favorable idea of him and of his position, and in that way might have saved many of his readers from doing him injustice. We cannot agree with him, however, so far as we can understand him, nor can we for a moment say anything in approval of the course he has pursued and advocated in the preparation of his work. We cannot see, and certainly have not as yet been shown, how the people are to be qualified to practice medicine, or to aid the practice, with an ill-digested and extremely superficial series of instructions in regard to the general character of certain diseases, injuries and conditions of the human frame, accompanied with similar instructions as to their general modes of treatment, and the application of special remedies—all comprised in one octavo volume of 725 pages. This single book, too, be it remembered, without a single line about anatomy, physiology, or the philosophy of health, is to prepare a man to appreciate the value of his medical attendant's prescription; in other words, to scrutinise and criticise, perchance to modify the latter's practice on himself and all his friends and neighbors.

We have no desire longer to abuse the good sense and patience of our readers by dwelling on this subject, nor do we feel called upon to enter upon a critical analysis of a work, whose whole tendency we are obliged to condemn. We therefore take our leave of Dr. Fort, with the hope that in the preparation of his next edition he will not, in his anxiety to teach the bedarkened public, so far lose sight of his strictly scientific friends, as to devote his evidently superior abilities and great experience to the production of another hybrid composition, of a class in which he has already been preceded by so many competitors, who will be only too happy to be honored with such respectable company.

